# Breathing silicon anodes for durable high-power operations

**DOI:** 10.1038/srep14433

**Published:** 2015-09-23

**Authors:** Chihyun Hwang, Sehun Joo, Na-Ri Kang, Ungju Lee, Tae-Hee Kim, Yuju Jeon, Jieun Kim, Young-Jin Kim, Ju-Young Kim, Sang-Kyu Kwak, Hyun-Kon Song

**Affiliations:** 1 School of Energy and Chemical Engineering, UNIST, Ulsan 689-798, Korea; 2 School of Materials Science and Engineering, UNIST, Ulsan 689-798, Korea; 3 KIST-UNIST Ulsan Center for Convergent Materials, UNIST, Ulsan 689-798, Korea; 4 GS Energy R&D Center, GS Energy Corp., Seoul 134-848, Korea; 5 KCC Corp., Seoul 137-703, Korea; 6 IBS Research Center for Multidimensional Carbon Materials, UNIST, Ulsan 689-798, Korea

## Abstract

Silicon anode materials have been developed to achieve high capacity lithium ion batteries for operating smart phones and driving electric vehicles for longer time. Serious volume expansion induced by lithiation, which is the main drawback of silicon, has been challenged by multi-faceted approaches. Mechanically rigid and stiff polymers (e.g. alginate and carboxymethyl cellulose) were considered as the good choices of binders for silicon because they grab silicon particles in a tight and rigid way so that pulverization and then break-away of the active mass from electric pathways are suppressed. Contrary to the public wisdom, in this work, we demonstrate that electrochemical performances are secured better by letting silicon electrodes breathe in and out lithium ions with volume change rather than by fixing their dimensions. The breathing electrodes were achieved by using a polysaccharide (pullulan), the conformation of which is modulated from chair to boat during elongation. The conformational transition of pullulan was originated from its α glycosidic linkages while the conventional rigid polysaccharide binders have β linkages.

Polysaccharides are omnipresent in nature. With a wide range of chemical and structural varieties, the biologically important polymers serve as energy storage materials^1^ (e.g., starch and glycogen), structural components of plants^2^ (e.g., cellulose and pectin) and extracellular materials bridging cells and surroundings^3^ (e.g., pullulan). Polysaccharides in organisms provide mechanical strength and adhesiveness^4^ while human beings have utilized starch as an energy source, cellulose^5^ as papers for information storage and some polysaccharides as glues for attachment.

Higher energy densities of energy storage devices such as lithium ion batteries (LIBs) have been pursued for longer uses of electrically powered machines from smart phones to electric vehicles^6^,^7^,^8^,^9^. Being overlapped with environmental issues, LIBs are attracting more attentions from our societies^10^,^11^. Silicon^12^,^13^,^14^, an anode material of LIBs, holds a lead in the competitive race of increasing capacity of the energy storage devices due to its high gravimetric capacity (~4200 mAh g^−1^) approximately 10 times as high as that of most widely used graphite^15^,^16^,^17^ (~370 mAh g^−1^). Moreover, its delithiation potential during discharge of batteries keeps lower than those of other high-capacity materials including conversion-reaction-based anode materials^18^,^19^: less than 0.6 V for silicon versus ~1 V for Co_3_O_4_.

Despite of the advantages, it is difficult to commercialize silicon anode materials due to large volume expansion and low ionic and electric conductivities^20^,^21^,^22^. During full lithiation, it expands up to 400% at room temperature. The volume change between lithiation and delithiation induces seriously significant stress leading to pulverization of silicon mass with the active material detached from current collector and then resulting in dramatic capacity fading with increasing cycles. At the same time, fresh surface of silicon is exposed to electrolyte with pre-developed solid-electrolyte interphase (SEI) layer broken during pulverization or crack development of electrodes so that extra electrons and electrolyte molecules are consumed for forming new SEI layer^12^,^23^,^24^,^25^. Various solutions have been proposed via incorporating buffer space into silicon morphology^13^,^26^,^27^,^28^,^29^,^30^, wrapping silicon with conductive or mechanically-rigid layers^31^,^32^,^33^,^34^ and using mechanically stiff binders^35^,^36^ to grab the active mass firmly. In this work, a binder solution is proposed to overcome the problems of silicon anode materials by providing strong adhesion to silicon as well as elasticity appropriate for composite electrodes.

Binders conventionally used for LIBs such as polyvinylidene fluoride (PVdF) cannot effectively keep integrity of composite electrodes containing silicon as an active material and conducting agents during repeated lithiation-delithiation cycles. Their weak adhesion to silicon as well as too elastic property to endure stress development during lithiation makes the binders inappropriate for the highly expandable anode material. Stiffer polymers were reported to be well matched with silicon. Silicon-based LIB cells gained enhanced capacity retention with alginate and carboxymethyl cellulose (CMC)^35^,^36^,^37^. With alginate as a binder, silicon cells delivered about 70% of stored energy when they were fully delithiated for about 40 min (2000 mAh g^−1^ of 3000 mAh g^−1^ at 1.5 C). 85% of the initial capacity was still usable after 100 cycles. On the other hand, PVdF and CMC experienced serious decay in capacity at the same condition. However, the CMC can work as an effective binder for silicon when it is cross-linked with polyacrylic acid (PAA). The stiffness of the mixed binder system, PAA/CMC, is tuned appropriately by more elastic or softer PAA. Also, the chemical linkages responsible for strong binding ability are formed between carboxylic groups of PAA and hydroxyl groups of surface oxide of silicon via condensation reaction at 150 °C.

From a chemical standpoint, the common thing of alginate and CMC is that they are polysaccharides containing β glycosidic linkages between constituent units or residues (Fig. 1a): D-glucose units or their derivatives are linked via β-(1 → 4) linkages to form cellulose or CMC; alginate is a copolymer of β-(1 → 4)-linked D-mannuronic acid and α-(1 → 4)-linked L-guluronic acid^36^. The β glycosidic linkages provide stiffer mechanical properties than their α counterparts^38^. Whether constituent pyranose rings can change conformationally from chair to boat during elongation determines the mechanical properties. Dextran containing α-(1 → 6) and amylose with α-(1 → 4) provide elasticity, experiencing the chair-to-boat transition with dimensional changes of the distance between neighboring glycosidic oxygens (O_i_-O_j_) from 0.44 nm to 0.57 nm in dextran and from 0.45 nm to 0.54 nm in amylose. However, cellulose based on β-(1 → 4) does not show the transition because the chair ^4^C_1_ conformer as its most stable form before elongation has the longest O_i_-O_j_ at 0.54 nm among all thermodynamically available conformations.

The already developed binders for silicon represented by PAA/CMC are too inelastic or stiff to accommodate strain developed during bending, folding and crumpling. Silicon-based electrodes with PAA/CMC experience severe cracking during lithiation even though the binder suppresses volume expansion with strong adhesion to the electroactive particles. More elastic properties are expected by replacing the β-linkage-based polysaccharide with a α-linkage-based one^39^. Pullulan (or α-1,4-;α-1,6-glucan), proposed as a component for novel binder systems in this work, is a polysaccharide polymer of α-(1 → 6)-linked maltotriose units (Fig. 1a)^40^. Within the maltotriose unit, three glucose residues are connected by α-(1 → 4) glycosidic bonds. The biopolymer, produced extracellularly by a yeast-like fungus Aureobasidium pullulan and localized on an outer surface of the chlamydospores, is non-toxic, edible and biodegradable with water-soluble but non-hygroscopic natures. Its structure can be described as an intermediate between amylose and dextran structures, considering co-existence of both α-(1 → 4)- and α-(1 → 6)-linkages.

As a novel binder system with more elastic properties, CMC (β polysaccharide) in PAA/CMC was replaced by pullulan (α polysaccharide) (Fig. 1b). Condensation reactions between carboxylate groups of PAA and hydroxyl groups of pullulan built an inter-linked network consisting of the two polymers. The heteropolymer inter-networking was confirmed by IR spectra (Fig. 1c)^35^. After heating the mixture of PAA and CMC at 150 °C, the -OH peaks of PAA and pullulan (broadly dispersed around 3,300 cm^−1^) disappeared in PAA/pullulan (left in Fig. 1c) while -COOH of PAA were converted to -COO- with a peak shift from 1,710 cm^−1^ to 1705 cm^−1^. Also, five membered rings including -OC-O-CO- fraction were observed at 1,805 cm^−1^, resulting from intra-condensation between –COOH of PAA. The networked or cross-linked heteropolymer binder system, PAA/pullulan, was insoluble in electrolytes used for LIBs (e.g. a mixture of ethylene carbonate (EC) and diethyl carbonate (DEC) at 3 to 7 in volume with 1.3 M LiPF_6_) at least for a period longer than several months while a physical mixture of the polymers kept soluble in the same condition. (Figure S1). Also, the PAA/pullulan was electrochemically stable without serious side reactions in a reductive potential window between 0 V and 1.2 V versus Li/Li^+^ (Figure S2).

Hardness and elastic modulus of PAA/pullulan (measured by nano-indentation; 40 μm-thick polymer films on cover glasses)^41^,^42^ were positioned between those of PAA/CMC and PAA as expected from the α linkage structure of pullulan (Fig. 2a and S3). The soft polymer PAA was hardened in terms of plastic deformation by hardness and also stiffened in terms of elastic deformation by elastic modulus when it was cross-linked by the polysaccharide binders, CMC or pullulan. Pullulan, relatively more elastic than CMC, led its mixture with PAA to be more elastic than PAA/CMC. The more elastic behavior of PAA/pullulan was easily confirmed in a macroscopic dimension scale by folding or crumpling the composite electrodes in which silicon active mass and conducting agents were bound together on copper current collectors by the help of binders. No cracks were developed with PAA/pullulan after severe folding while macroscopic cracks were seriously introduced to the PAA/CMC-based electrodes (Fig. 2b). In a single-molecular (or monomer) level elongation simulation, conformation of pyranose units changed from chair to boat in α-(1 → 4) (e.g., amylose) with 20 kcal mol^−1^ as the activation energy for the transition and α-(1 → 6) (e.g., dextran) with 25 kcal mol^−1^ (Fig. 2c)^43^,^44^. In the trimer-level elongation simulation considering two different ensembles resulting from combination of α-(1 → 4) and/or α-(1 → 6) as possible fragmentation models of pullulan, the chair-to-boat transitions were developed along elongation (Figure S4a,b). All-atom molecular dynamics simulation by using the COMPASS forcefield^45^ also showed the same trend of mechanical properties in that pullulan is more elastic than CMC in stress-strain curves (Fig. 2d). During the double elongation, configurational change to the boat form (indicated by yellow in Fig. 2e) of pyranose rings as well as de-folding of polymeric backbones relaxed the stress of stretching in pullulan (30 of 10mers). However, CMC did not experience such a transition on elongation in both the monomer-, trimer- and polymer-level simulations (Fig. 2c, S4d and 2e).

The mechanical properties of polysaccharide components of binder systems significantly affected electrochemical properties of silicon-based anodes. Capacities of silicon-based electrodes were compared between PAA/pullulan as the more elastic polysaccharide binder and PAA/CMC as the conventional one. For establishing stable solid-electrolyte interphase (SEI) layer, silicon anodes in half cells with lithium metal were galvanostatically lithiated and then delithiated at 0.05 C as a slow rate. Both aqueous and non-aqueous solvents (N-methyl-2-pyrrolidone or NMP) were possible for dispersing PAA/pullulan so that composite slurries containing silicon, carbon black and the binder were made in either solvent. However, PAA/CMC is dispersed not in NMP but in water. The initial coulombic efficiencies (η_o_) of PAA/pullulan-based cells were 84% (1863 mAh g^−1^/2215 mAh g^−1^) with aqueous solvent and 77% (1936 mAh g^−1^/2493 mAh g^−1^) with NMP (Figure S5). The reversible capacities at the cycles following the first cycle were similar between the aqueous and organic dispersion solvents. The values of η_o_ are comparable to that of PAA/CMC cells (η_o_ = 81% = 1,893 mAh g^−1^/2,331 mAh g^−1^). Except of η_o_, all electrochemical characteristics of the PAA/pullulan cells such as rate capability and cyclability were observed closely similar independent of solvents used for dispersion (Figure S6).

The capacity retention with cycles was significantly enhanced at slow lithiation and delithiation rates (0.2 C and 0.5 C, respectively) by the use of PAA/pullulan (Fig. 3a,b). Cells based on PAA and PAA/CMC did not work after 100 and 150 cycles respectively. PAA did not endure volume change of silicon mass due to its lack of stiffness so that PAA cells did not work after 100 cycles. Stiffer mechanical properties of polysaccharides enabled cells of PAA/CMC and PAA/pullulan to deliver a meaningful capacity at the 100^th^ cycle (76% and 86% of the initial capacity, respectively). The average coulombic efficiency (η_avg_) of PAA/pullulan cells after the initial cycle to the 100^th^ cycle was ~100%, which was higher than that of PAA/CMC cells (98.4%) (Figure S7). Also, potential profiles indicate that higher overpotential was developed with PAA/CMC than PAA/pullulan after the 100^th^ cycle (Fig. 3a and S8). After 150 cycles, however, PAA/CMC-based electrodes did not keep the capacity due to its too high stiffness or rigidity, developing cracks to disperse stress caused by severe volume change (confirmed by morphological investigation below in Fig. 4a). Our PAA/pullulan showed good cyclability up to the 200^th^ cycle, reaching 78.6% of initial capacity at the cycle. Moreover, electrodes of higher silicon contents at 80 wt.% silicon with 10 wt.% PAA/pullulan and carbon black (cf. 60 wt.% silicon was used with 20 wt.% of binder and carbon black in Fig. 3) also demonstrated such a good cycling performance comparable to the 60 wt.% silicon cells (Figure S9). Due to its elasticity based on chair-to-boat transition, PAA/pullulan appears likely to breathe in and out reversibly on lithiation (expansion) and delithiation (contraction), keeping a certain degree of porosity of electrodes without generating cracks or losing contact to electric pathways (confirmed by morphological investigation below in Fig. 4a).

In addition to stable capacity retention during cycles, delithiation kinetics was also enhanced with our PAA/pullulan (Figure S10; the current rates for lithiation and delithiation are fixed at the same value.) probably due to its maintaining porous structure (Fig. 4a). Its delithiation capacities were estimated higher than those of PAA/CMC especially at higher current rates even if the capacities at 0.2 C are similar between the two binder systems. The kinetic gains by our binder are more emphasized when considering delithiation capacities of cells that was lithiated at a slow rate, 0.2 C (Fig. 3c,d). Surprisingly, there were no change in capacity from 0.5 C to 30 C (the rate at which stored energy is completely extracted for only 2 min.) with PAA/pullulan while the capacity of PAA/CMC cells fell down to ca. 25% of the capacity at 0.5 C. Even at 50 C, the capacity around 1,000 mAh g^−1^ is available with our binder. Smaller charge transfer resistance (R_CT_) as well as larger diffusion coefficient (D) confirms the faster processes via PAA/pullulan: R_CT_ = 4.4 ohm and D = 4.54 × 10^−14^ cm^2^ sec^−1^ for PAA/pullulan; R_CT_ = 10.9 ohm and D = 3.51 × 10^−14^ cm^2^ sec^−1^ for PAA/CMC (calculated from impedance spectra shown in Figure S11)^46^.

Excellent cyclability at fast lithiation/delithiation can be expected for our PAA/pullulan from its enhanced cyclability and rate capability discussed above. The remarkable differences of capacity retention between PAA/pullulan and PAA/CMC cells were observed at 6 C/6 C as lithiation/delithiation rates without potentiostatic period during lithiation (Fig. 3e). The current rates indicate that electric vehicles can be charged within 10 min and its speed can be accelerated at the power at which energy stored in batteries is fully extracted within 10 min. Such a tough operation decreased cycle life of the PAA/CMC cell tremendously: its capacity decayed to 50% of the initial value after 100 cycles. Incomparable to its counterpart, however, our PAA/pullulan demonstrated much more improved cyclability showing 57% of the initial capacity at the 1,000^th^ cycle.

We traced what happens inside cells during lithiation and delithiation by investigating morphological changes of electrodes (Fig. 4). Both PAA/pullulan and PAA/CMC electrodes were prepared with the same morphological texture and thickness at 10 um before lithiation (Fig. 4a). By a simple calculation based on densities of electrodes and their components and thickness of electrodes before and after lithiation (calculation section in Supporting information), volume fraction of each component in electrodes before lithiation was estimated at 25.8% for silicon (**V**_**1**_^**o**^), 22.2% for binder and carbon black (**V**_**2**_^**o**^) and 52.0% for void (**V**_**3**_^**o**^). After full lithiation of the electrode based on PAA/CMC, volume expansion of silicon densified the electrode texture and eliminated the voids between particles existing before lithiation, decreasing electrode porosity (ε) from 52% to 30% and increasing thickness from 10 um to 18 um. However, the absolute value of void volume was not changed even if the porosity decreased: void volume expansion coefficient (β) = 1.0 (Fig. 4b). The most prominent feature was macroscopic cracks induced by lithiation-delithiation cycles (Figure S12). On the other hand, the electrode based on our PAA/pullulan kept the initial porous morphology even after lithiation (Fig. 4a; ε = 52% to 46% with a small decrease in Fig. 4b). Its void volume increased twice (β = 2.0) on lithiation, which results from more resilient and less stiff properties of pullulan. Inevitably, volume expansion of silicon led to larger thickness change from 10 um to 23 um (50% point larger than that of PAA/CMC). However, due to the breathing behavior of electrode supported by pullulan, there were no cracks (or severe pulverization) observed. The strong adhesion of PAA/pullulan to current collectors prevented the composite electrode layer from being detached from current collectors (Figure S13; peel strength = 2.7 N mm^−1^ for PAA/pullulan versus 0.35 N mm^−1^ for PAA/CMC).

In a summary of this work, four property domains are logically connected in a sequential way: molecular-level conformation of polysaccharides, mechanical properties of the polymers, morphological characteristics of electrodes and electrochemical performances of cells. β-linked polysaccharides (CMC in this work) characterized by high hardness and modulus, showing no conformational change during elongation, limited the volume expansion of silicon-based electrodes during lithiation so that electrodes were densified with less porosity. In the situation, only way to release the developed stress was for electrodes to be cracked. Such a severe deformation of electrodes led to poor electrochemical kinetics and stability. On the other hand, α-linked polysaccharides (pullulan in this work) showed more elastic and softer mechanical characteristics, which resulted from the chair-to-boat conformation on elongation in a monomeric unit level. Synchronizing with electrochemical lithiation and delithiation, the electrodes based on the α-linked polysaccharide breathe in and out with volume expansion and contraction of silicon mass. The most emphasized point is that the integrity of electrodes was completely secured without any cracks and/or fragmentation. PAA and PVdF as more elastic binders cannot support the electrode integrity. Resultantly, the cells based on electrodes using the α-linked polysaccharide as a binder showed enhanced electrochemical performances especially at more severe charging/discharging conditions.

## Methods

### Binder preparation

A 10 wt.% binder solution of PAA/pullulan was prepared. PAA (Sigma-Aldrich, Average Mw = 250,000) and pullulan (Hayashibara, Average Mw = 200,000) at the weight ratio of 8:2 were dispersed in water or NMP. The solution was stirred at 90 °C for 3 h and cooled down gradually. As a control, a 10 wt.% binder solution of PAA/CMC was prepared by mixing PAA and CMC (Sigma-Aldrich, average Mw = ~250,000) at the weight ratio of 5:5.

### Cell preparation

2032 coin-type half cells were assembled with a silicon-based composite electrode as a working electrode and lithium metal as a counter electrode. A porous separator (NH716, Asahi) was sandwiched between the working and counter electrodes. For the working electrode, a mixed slurry of silicon nanoparticles (npSi; unless otherwise indicated; Sigma Aldrich, 100 nm size; Figure S14), binder and carbon black (Super P, Timcal) at the 6:2:2 weight ratio was coated on copper current collector by doctor blade. Loading density of the slurry was ~1 mg cm^−2^ with thickness at 10 to 15 um. PAA/CMC, PAA or PAA/pullulan was used as the binder. Electrolyte was 1.3 M LiPF_6_ solution in a mixture of ethylene carbonate (EC) and diethyl carbonate (DEC) at 3:7 volume ratio with 10 wt.% fluoroethylene carbonate (FEC).

### Mechanical characterization

Nano-indentation tests (XP module in Agilent G200) were carried out using three-sided pyramidal diamond Berkovich indenter. Maximum indentation depth was 1000 nm while loading rate was the constant indentation strain rate at 0.05 s^−1^. Hardness and elastic modulus as a function of indentation depth were obtained by the continuous stiffness measurement (CSM). Hardness and elastic modulus were analyzed at indentation depth >200 nm to remove the effects of imperfect calibration of indenter tip geometry and the effects of sample surface.

### Monomer and trimer elongation simulation

Single molecule elongation was simulated to identify the elasticity and conformational transition of glucopyranose ring in pullulan and CMC under strain in detail. The structures of α-D-glucose and β-D-glucose were obtained by conformational analysis. All possible conformations of glucopyranose ring were generated by using systematic grid scan with torsion angle at an interval of 10 degree in all torsion angle range. Resulting structures were optimized with the COMPASS forcefield. The cut-off distance 1.85 nm was used for electrostatic and van der Waals interactions. The most stabilized structures of α-D-glucose and β-D-glucose followed ^4^C_1_ conformation with energy of 31.01 kcal mol^−1^ and 34.02 kcal mol^−1^, respectably. Those two molecules were equilibrated at 274.15 K using molecular dynamics (MD) simulation with the canonical (i.e. *NVT*) ensemble for 5 ns with Berendsen thermostat after constraining an oxygen atom in one end of molecule (i.e. O_1_) and another oxygen in the other end of molecules (i.e. O_4_ or O_6_) depending on the direction of elongation. Single molecule elongation simulation was run at the isothermal condition in such a way that O_1_ atom was pulled in the direction parallel to the line connecting two oxygen atoms at constant velocities of 10^−7^ and 10^−8^ nm fs^−1^. The position of O_1_ was updated every 1000 fs.

### Polymer system elongation simulation

All-atom molecular dynamics (MD) simulations were performed by using the COMPASS forcefield with a time step of 1 fs. Electrostatic and van der Waals interactions were calculated by PPPM (Particle-Particle Particle-Mesh) summation and atom-based methods, respectively. Periodic boundary condition was applied in three dimensions. For the uniaxial elongation simulation, the polymer systems were constructed by packing model polymers (i.e. 120 of 25 mers for PVDF, 30 of 10 mers for pullulan, and 30 of 30 mers for CMC) randomly into a cubic box in such a way that density was approximately 1 g cm^−3^. We stabilized the system by running *NVT* and *NPT* (i.e. isobaric-isothermal) MD simulations for 100 ps each and repeated this process three times at 428.15 K and 1 atm with Nose thermostat and Berendsen barostat to control temperature and pressure, respectively. Additional *NPT* MD simulation was performed for 500 ps, which was long enough to exhibit a stable density. Then, the system was cooled down to 298.15 K at constant pressure with a cooling rate of 0.65 K ps^−1^ and then the final system was run by *NPT* MD simulation for 500 ps at 1 atm and 298.15 K. The resulting system was used for the initial configuration of the isometric uniaxial elongation simulation. The non-equilibrium MD was done in such a way that the box length was increased every 0.1 ps in accordance with a strain rate of 10^10^ s^−1^, which doubles the box length at 100 ps.

### Electrochemical characterization

All cells were charged and discharged galvanostatically (WBSCS, WanATech). Cyclic voltammograms and impedance spectra were measured by a potentiostat equipped with an impedance spectroscopy (VMP3, BioLogic).

## Additional Information

**How to cite this article**: Hwang, C. *et al.* Breathing silicon anodes for durable high-power operations. *Sci. Rep.* **5**, 14433; doi: 10.1038/srep14433 (2015).

## Supplementary Material

Supplementary Information

## Figures and Tables

**Figure 1:**
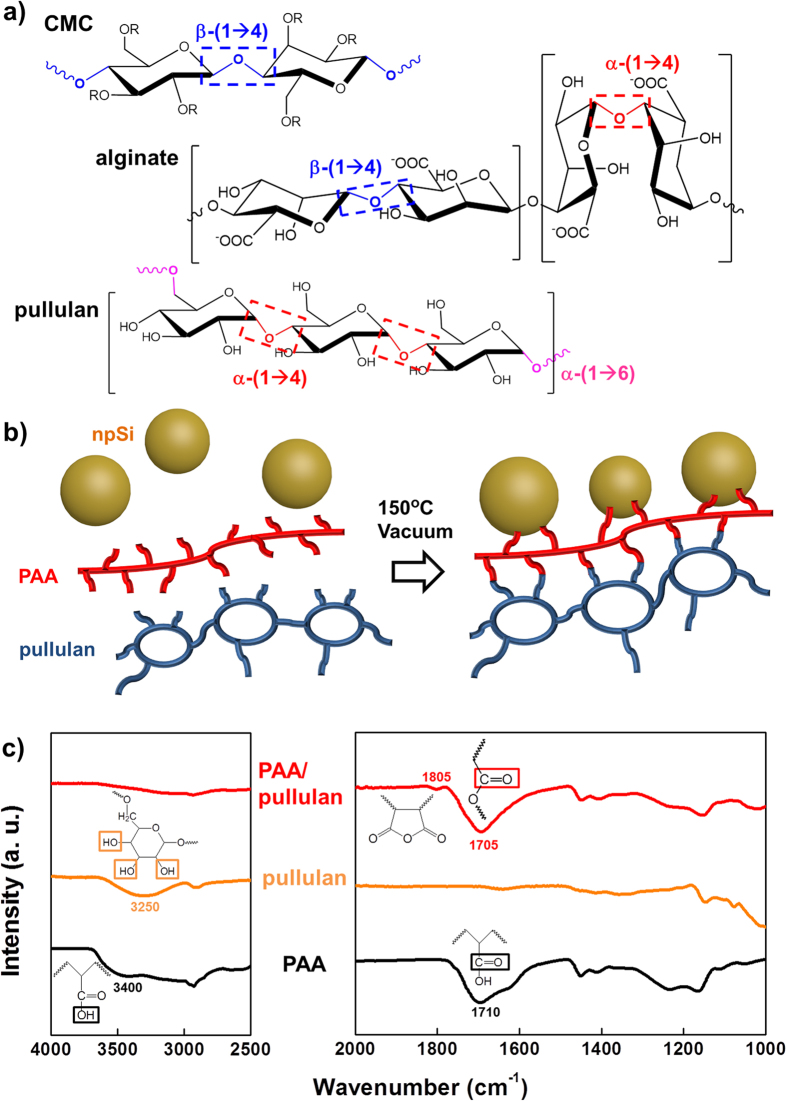
Chemical identification. (**a**) Molecular structure of three different polysaccharides: CMC, alginate and pullulan. Types of glycosidic linkages were indicated. (**b**) Covalent inter-linking between npSi and PAA as well as between PAA and pullulan after thermal treatment at 150 °C. Condensation reactions are induced at the temperature between carboxylic groups of PAA and hydroxyl groups of pullulan and surface oxide of silicon. (**c**) Infrared spectra confirming the covalent inter-linking by hydroxyl group disappearance and ester group generation.

**Figure 2:**
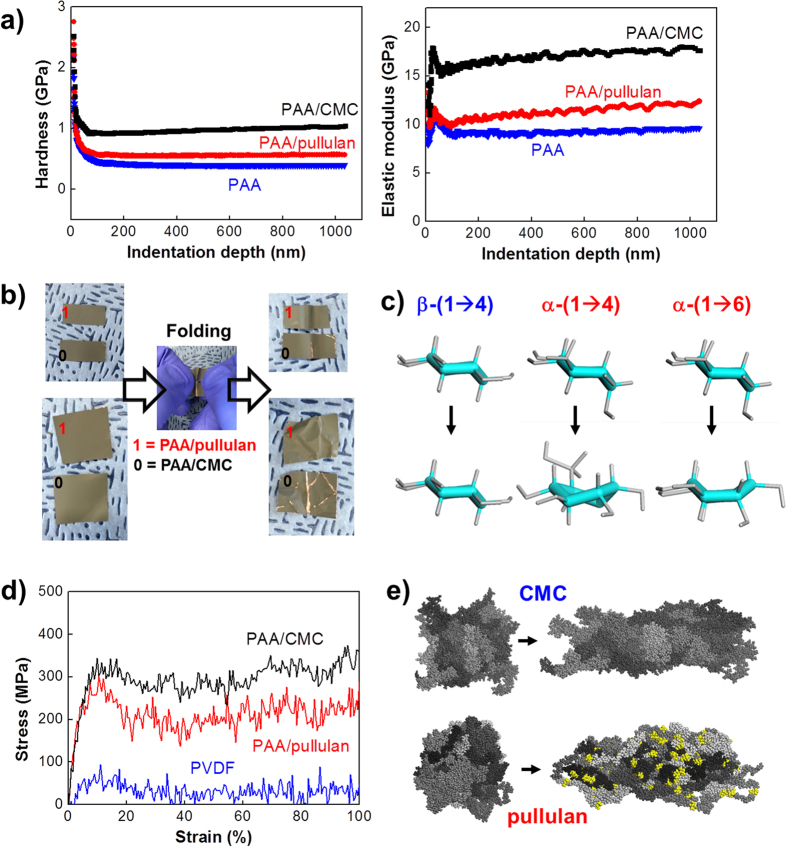
Mechanical properties and related molecular configuration. (**a**) Hardness and elastic modulus of three different binder systems characterized by nanoindentation: PAA, PAA/CMC and PAA/pullulan. (**b**) Macroscopic crack development on silicon-based electrodes after folding and crumpling. The electrodes were made of npSi, a binder and carbon black in 6:2:2 weight ratio (loading density = ~0.8 mg cm^−2^). PAA/pullulan or PAA/CMC was used as the binder. (**c**) Molecular-level conformational changes of pyranose units of different glycosidic linkages during elongation. β-(1 → 4) represents CMC while α-(1 → 4) and α-(1 → 6) represent pullulan. Chair-to-boat conformation was found in α cases while the conformation was fixed at chair for β case even after elongation. (**d**) Simulated stress-strain curves for PVDF (120 of 25 mers), pullulan (30 of 10 mers) and CMC (30 of 30 mers). The maximum strain represents double elongation of original systems. (**e**) Chair-to-boat conformational changes in polymer system level. Pullulan (30 of 10 mers) and CMC (30 of 30 mers) were doubly elongated in one direction. The initial and final states were presented on the left and right, respectively. A polymer chain was distinguished from another by a different scale of grey. The pyranose in boat conformation was indicated by yellow.

**Figure 3:**
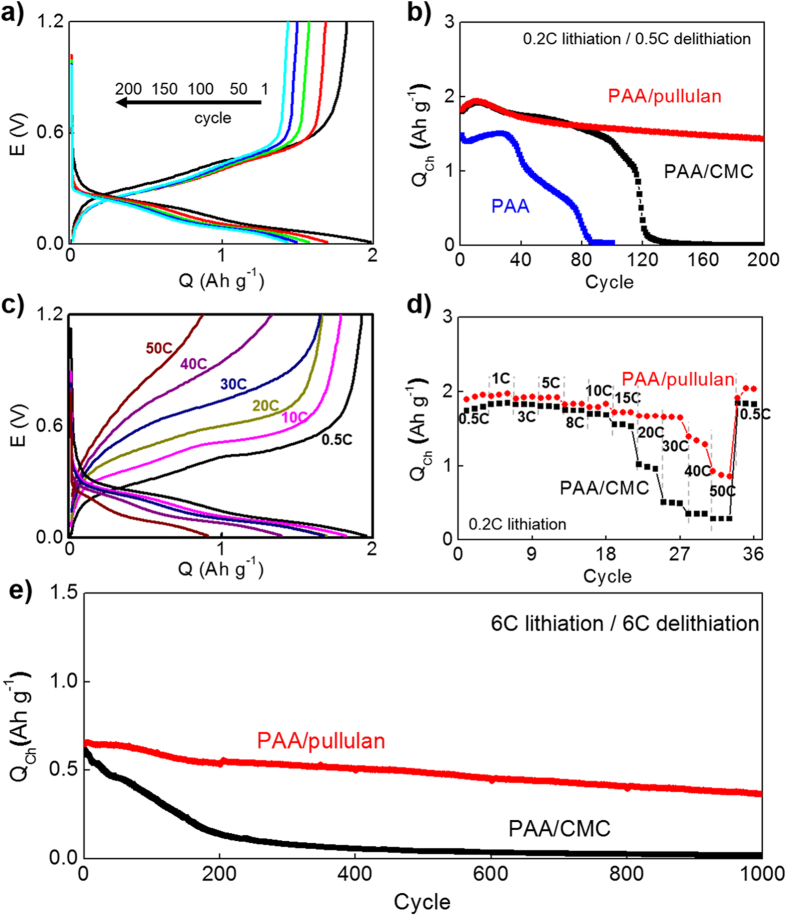
Electrochemical characterization. (**a**) Potential profiles of a coin-type half-cell containing an electrode of silicon and carbon black with PAA/pullulan as a binder on lithiation and delithiation at representatively selected cycles. Each cycle consisted of lithiation followed by delithiation. The cell was galvanostatically lithiated at 0.2 C as well as delithiated at 0.5 C in a potential range of 0.01 V to 1.2 V. (**b**) Delithiation capacities of three different binder systems as cycle progress. The same operational condition was used as in a. (**c**) Potential profiles of a PAA/pullulan cell at different delithiation rates with a fixed lithiation rate at 0.2 C. (**d**) Delithiation capacities of PAA/pullulan and PAA/CMC-based cells at different C rates. (**e**) Capacity retention with high-rate cycles consisting of 6 C lithiation followed by 6 C delithiation without potentiostatic retention. Capacities were calculated as per silicon mass.

**Figure 4:**
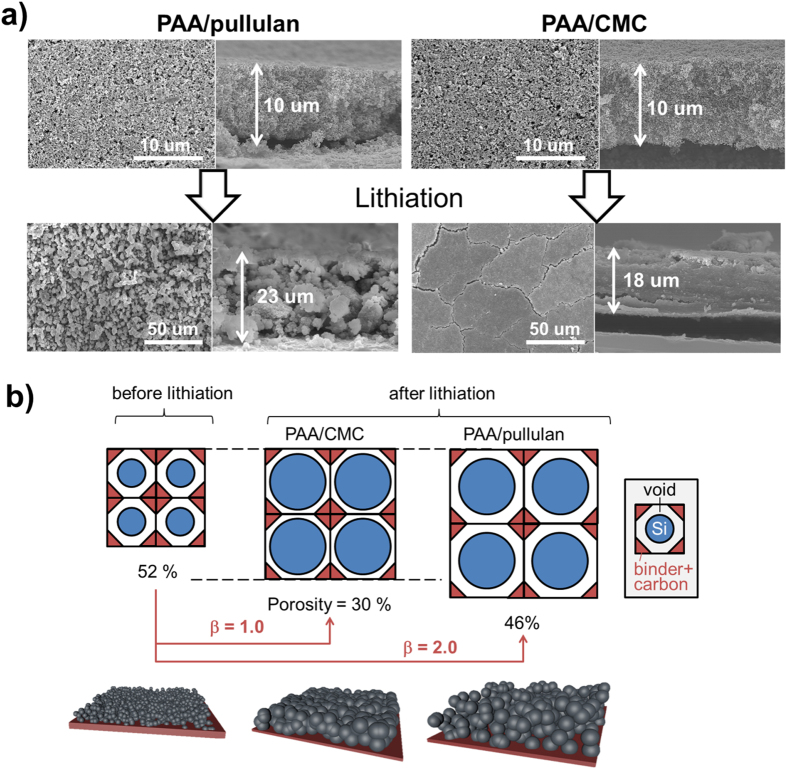
Morphological and dimensional changes on lithiation. (**a**) Electron-microscopic snapshots of silicon-based electrodes in top and cross-sectional views before (top) and after (bottom) lithiation. PAA/pullulan and PAA/CMC-based electrodes were compared. (**b**) 2D schematic description of morphological and dimensional changes on lithiation. Each square consists of three categorized components: npSi (blue circle), binder + carbon black (red triangle) and void (the rest part with no color). The area of each component represents its volume fraction. The connectivity among npSi, binder and carbon black was not considered. Porosity and void volume expansion coefficient (β = void volume ratios of before to after lithiation) were indicated. Also, 3D cartoon corresponding to each 2D description was demonstrated at the bottom.
