# Supplementary material for: Breathing silicon anodes for durable high-power operations
Source: Sci Rep. 2015 Sep 23;5:14433. doi: 10.1038/srep14433 (PMC4585771; doi:10.1038/srep14433)
Supplement: Supplementary Information [file srep14433-s1.pdf]

Supporting information

Breathing silicon anodes for durable high-power operations

Chihyun Hwang,[1] Sehun Joo,[1] Na-Ri Kang,[2,3] Ungju Lee,[4] Tae-Hee Kim,[1] Yuju Jeon,[1] Jieun Kim,[1] Young-Jin Kim,[5] Ju-Young Kim,[2,3,6,*] Sang-Kyu Kwak,[1,*] and Hyun-Kon Song[1,*]

[1]School of Energy and Chemical Engineering, UNIST, Ulsan 689-798, Korea

[2]School of Materials Science and Engineering, UNIST, Ulsan 689-798, Korea

[3]KIST-UNIST Ulsan Center for Convergent Materials, UNIST, Ulsan 689-798, Korea

[4]GS Energy R&D Center, GS Energy Corp., Seoul 134-848, Korea

[5]KCC Corp., Seoul 137-703, Korea

[6]IBS Research Center for Multidimensional Carbon Materials, UNIST, Ulsan 689-798, Korea

[*]E-mail: juyoung@unist.ac.kr, skkwak@unist.ac.kr, philiphobi@hotmail.com

Calculation on volume fraction of each categorized constituent of electrode

■ Definition

	Before lithiation	After lithiation
1 = Si	V_1^o	$V_1 = \alpha V_1^o$
2 = binder + carbon black	V_2^o	$V_2 = V_2^o$
3 = Void	V_3^o	$V_3 = \beta_i V_3^o$
tot = total	$V_{tot}^o = V_1^o + V_2^o + V_3^o = 100$	$V_{tot}^i = V_1 + V_2 + V_3$ $= \alpha V_1^o + V_2^o + \beta_i V_3^o$

α = volume expansion coefficient of Si =4

β_i = volume expansion coefficient of void

i = binder : i = CMC for PAA/CMC; i = pull for PAA/pullulan

■ Equations

○ From thickness of electrodes before and after lithiation.

$$V_1^o + V_2^o + V_3^o = V_{tot}^o \ \ (V_{tot}^o = 100) \qquad (1)$$

$$\alpha V_1^o + V_2^o + \beta_{CMC} V_3^o = V_{tot}^{CMC} \ \ (V_{tot}^{CMC} = 180) \qquad (2)$$

$$\alpha V_1^o + V_2^o + \beta_{pull} V_3^o = V_{tot}^{pull} \ \ (V_{tot}^{pull} = 230) \qquad (3)$$

○ From electrode composition

$$\frac{m_2}{m_1} = \frac{d_2 V_2^o}{d_1 V_1^o} \Rightarrow \frac{V_2^o}{V_1^o} = \left(\frac{m_2 d_1}{m_1 d_2}\right) = \gamma \qquad (4)$$

with d = density: $d_1 = 2.32$ g cm^{-3}; $d_2 \sim 1.8$ g cm^{-3}; m = mass: $m_1 = 6$; $m_2 = 4$

○ From loading density d_{ED} (ED = electrode)

$$d_{ED} = \frac{m_{ED}}{V_{ED}} = \frac{d_1 V_1^o + d_2 V_2^o}{V_1^o + V_2^o + V_3^o} \qquad (5)$$

with d_{ED} = 1g cm^{-3}

○ 5 unknowns ($V_1^o, V_2^o, V_3^o, \beta_{CMC}, \beta_{pull}$) can be determined from the 5 equations.

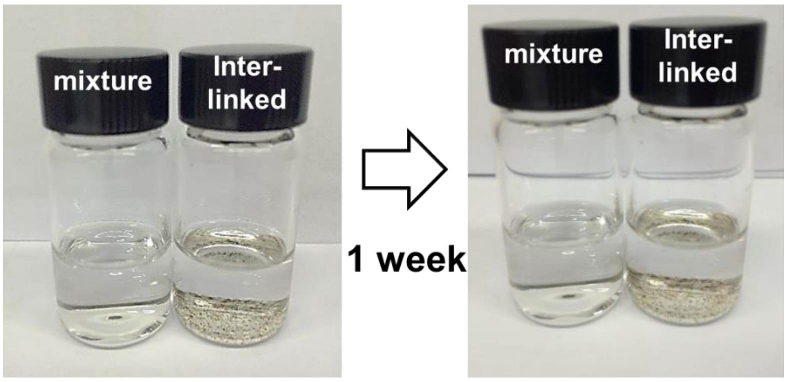

Figure S1 | Solubility in an electrolyte and water. Shown are a physical mixture of PAA and pullulan (mixture) and the thermally inter-linked product of them (inter-linked or PAA/pullulan) in an electrolyte (1.3M $LiPF_6$ in EC:DEC (3:7 v/v) + 10 wt. % FEC). The snapshots were taken just after and a week after introducing the polymers into the electrolyte. The physical mixture was completely soluble at least for one week while the inter-linked PAA/pullulan existed as an insoluble precipitate for the same time period.

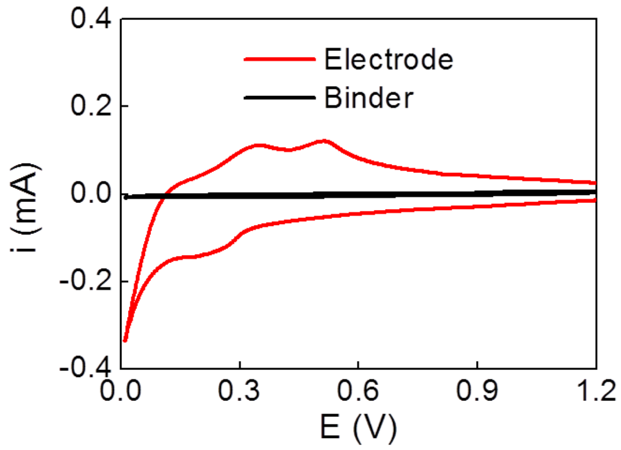

Figure S2 | Electrochemical stability. Cyclic voltammograms of PAA/pullulan (black) and an anode based on a mixed composite of npSi and carbon black in presence of PAA/pullulan (red). The PAA/pullulan film was solution-casted on a copper current collector. The same anode used in cells for electrochemical characterization was used. Significant amount of current ascribed to considerable electrochemical side reactions was not found on PAA/pullulan.

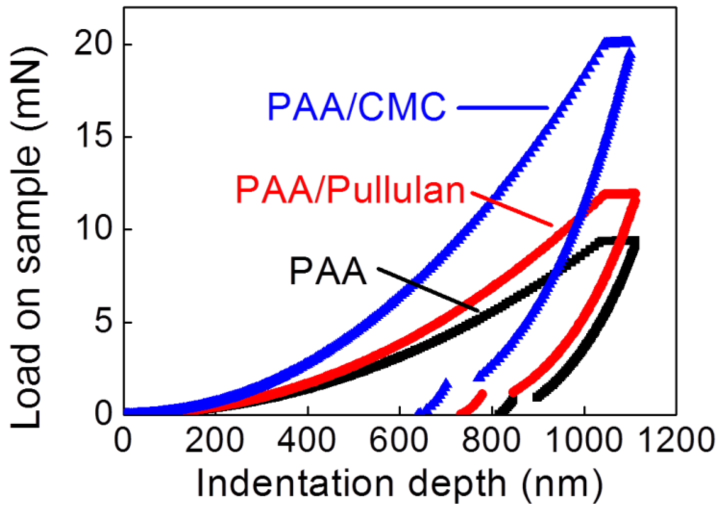

Figure S3 | Force-displacement curves by nanoindentation.

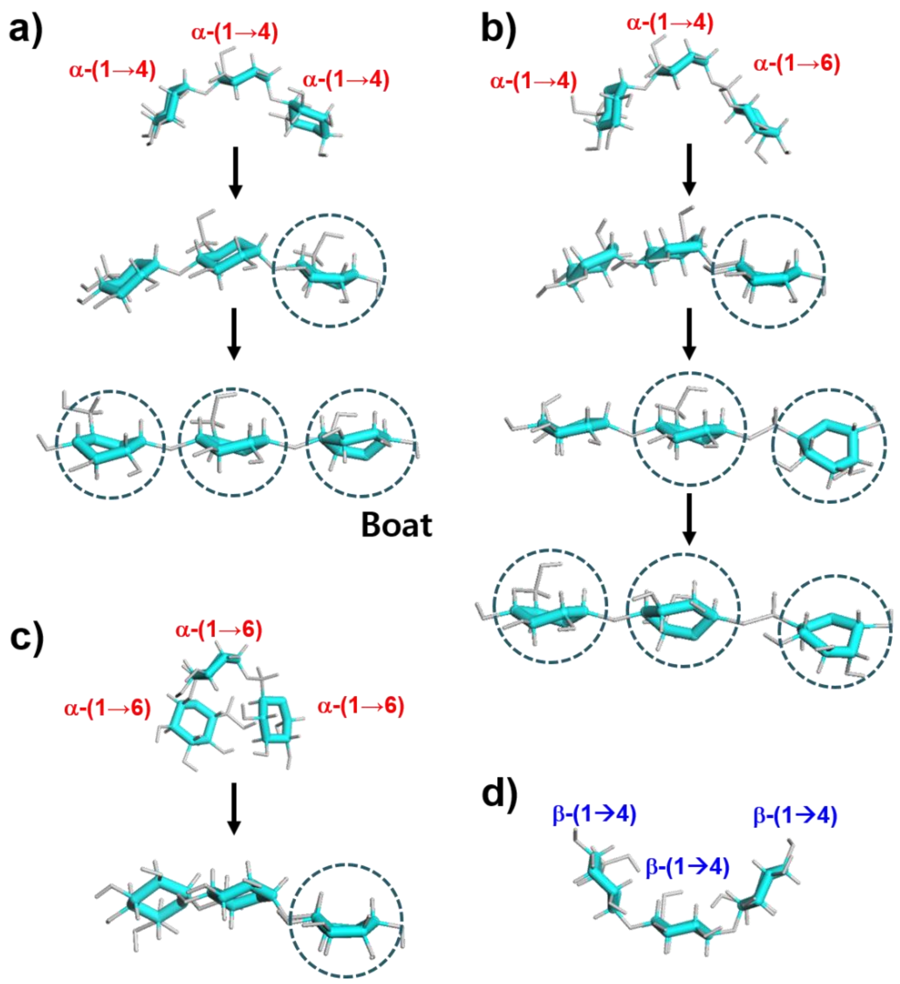

Figure S4 | Configurational changes of trimers during elongation by molecular dynamics simulation. **a** to **c)** Three different ensembles resulting from combination of α-(1→4) and α-(1→6) linkages. **a** and **b** represent fragmentation models of pullulan. **d**) A fragmentation model of CMC without chair-to-boat transition during elongation. Dashed circles indicate the boat form of pyranose unit.

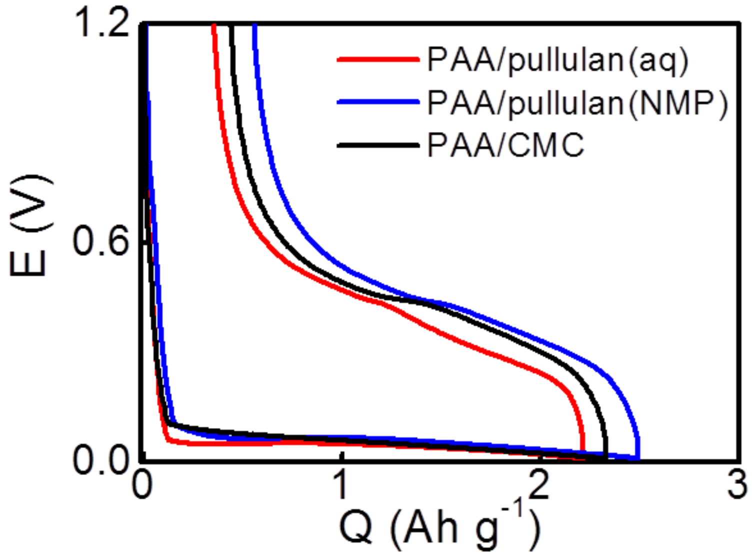

Figure S5 | Potential profiles at the first galvanostatic charge and discharge.

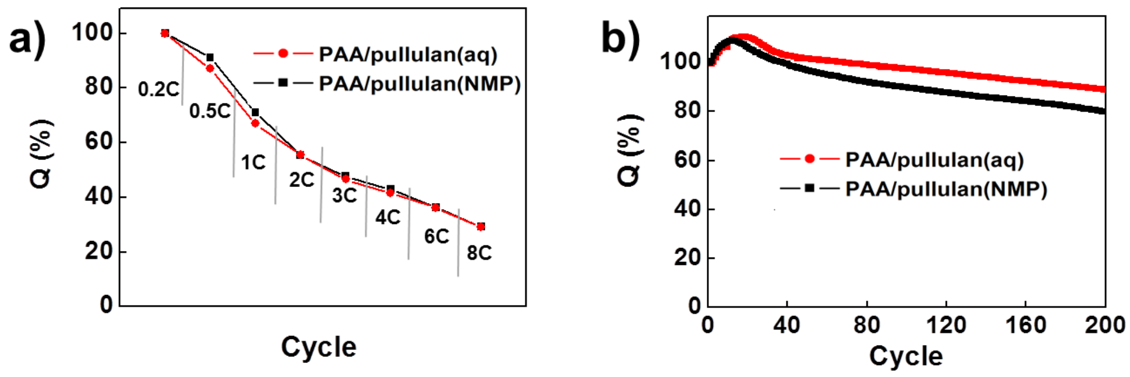

Figure S6 | The effects of dispersion solvents on electrochemical performances of silicon anode cells based on PAA/pullulan as binder. a) Rate capability. **b**) Cyclability at 0.2C lithiation and 0.5C delithiation. Water or NMP (N-methyl-2-pyrrolidone) was used as the dispersion solvents for making composite slurries including silicon, binder and conducting agents.

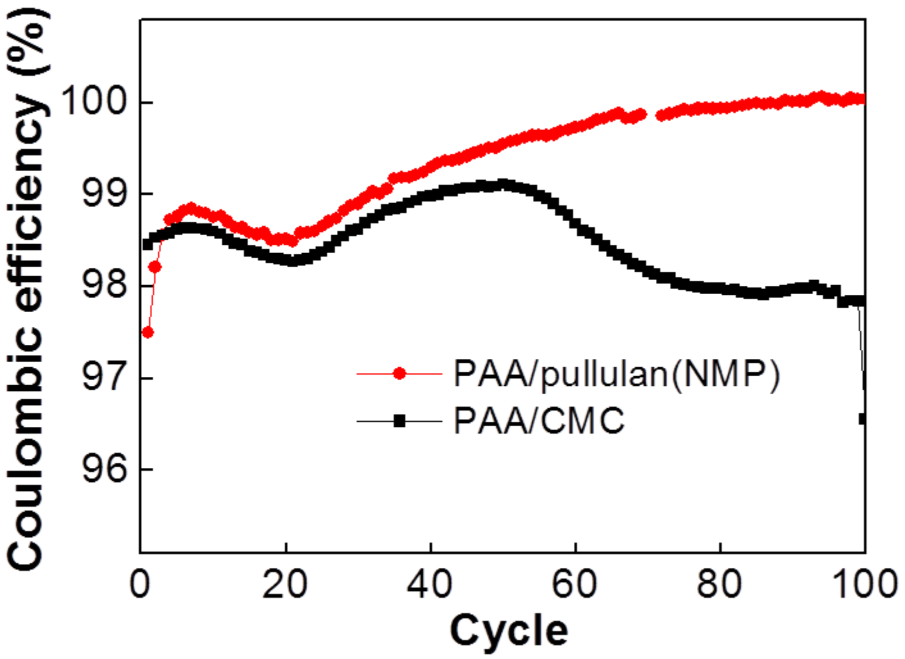

Figure S7 | Higher columbic efficiencies with PAA/pullulan during cycling at 0.2C lithiation and 0.5C delithiation.

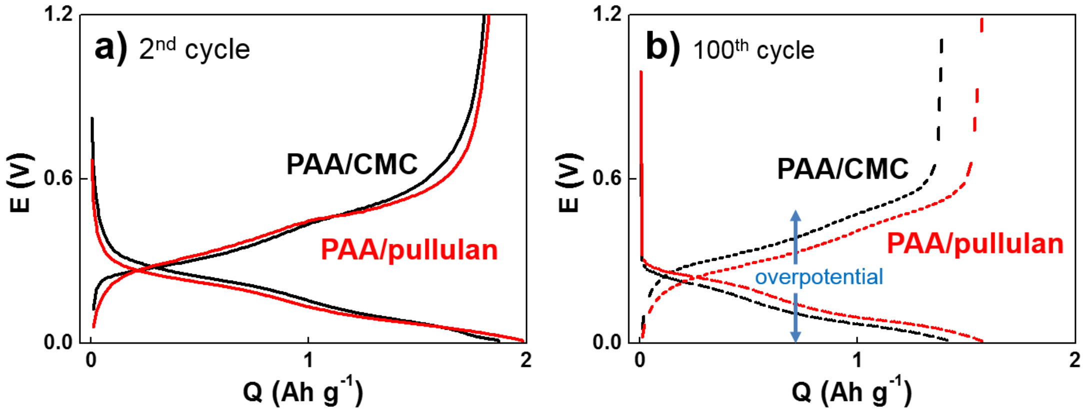

Figure S8 | Smaller overpotential development with PAA/pullulan after cycling at 0.2C lithiation and 0.5C delithiation. a) Potential profiles at the 2nd cycle. **b**) Potential profiles at the 100th cycles.

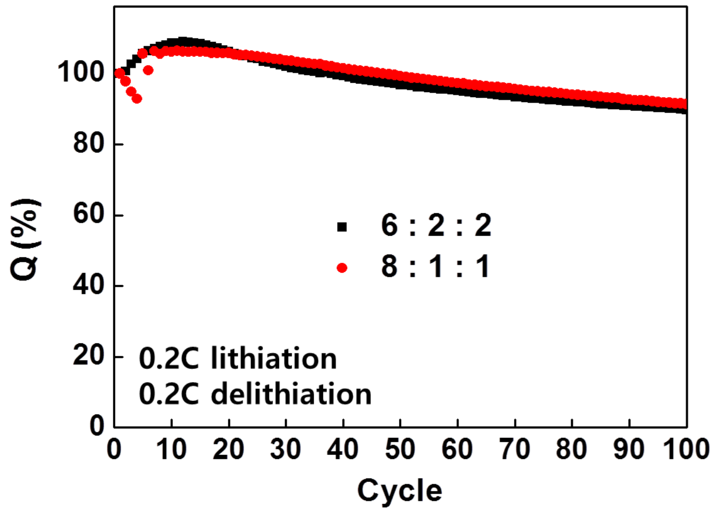

Figure S9 | Cyclability of high-loading cells with PAA/pullulan. The electrode of higher silicon contents at 80 wt. % silicon with 10 wt. % PAA/pullulan and carbon black (8:2:2) is comparable to the 60 wt. % silicon cells (6:2:2, 60 wt. % silicon with 20 wt. % of binder and carbon black) in terms of cycle performances.

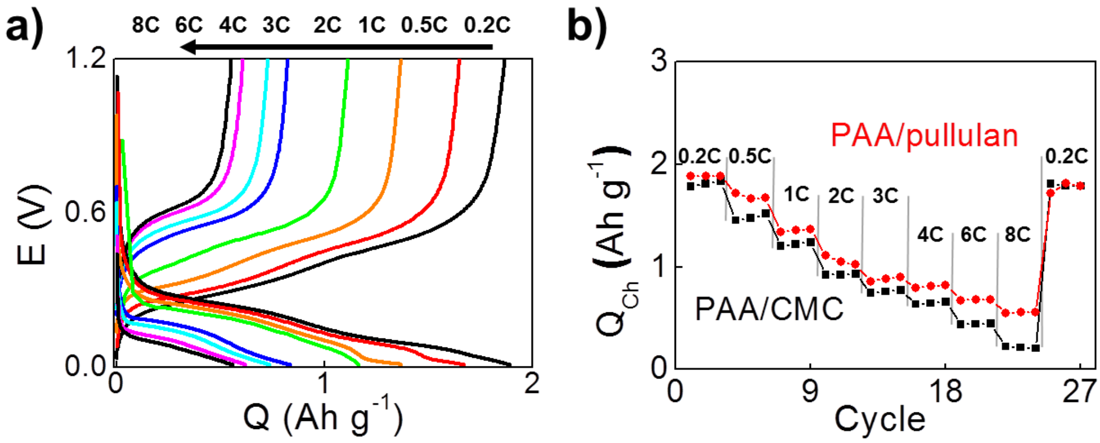

Figure S10 | Comparison of charge and discharge rate capability at same time. (a) was shown voltage profile of PAA/pullulan binder electrode at each rate. (b) indicates capacity of PAA/pullulan and PAA/CMC binder electrode in accordance with rate.

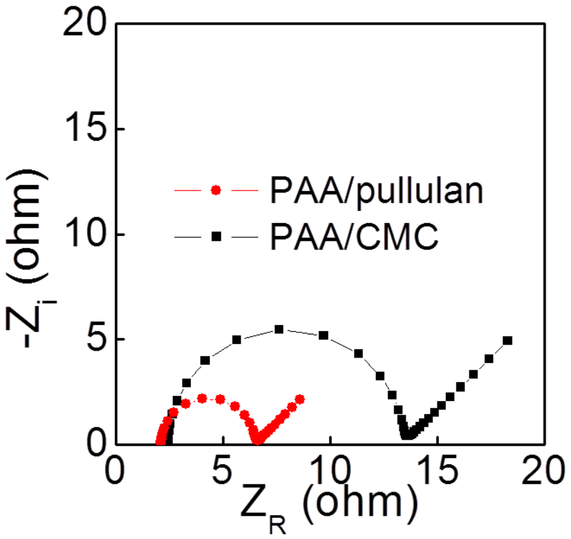

Figure S11 | Electrochemical impedance spectra of silicon anode cells based on PAA/CMC and PAA/pullulan. The impedance data obtained in a frequency range of 200 kHz to 100 mHz after full lithiation were fitted by the equivalent circuit of Randles.

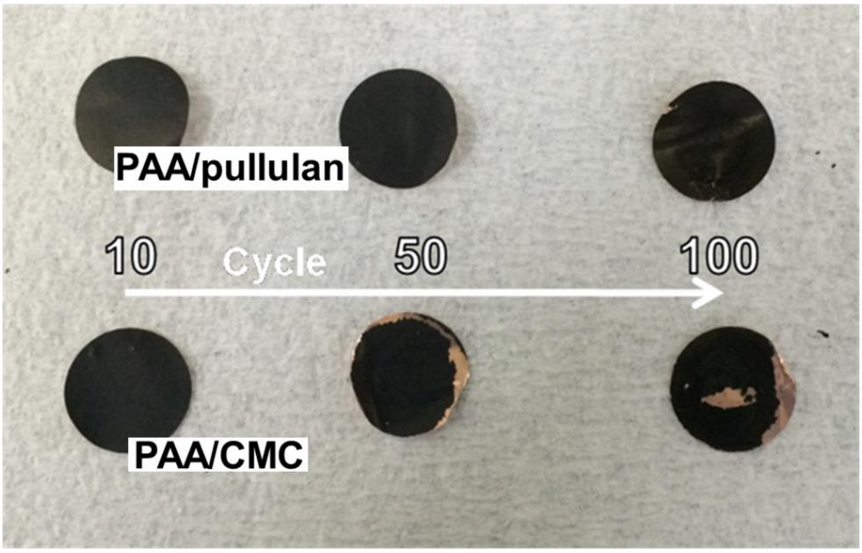

Figure S12 | Electrode integrity of silicon anodes experiencing 10, 50 and 100 cycles.

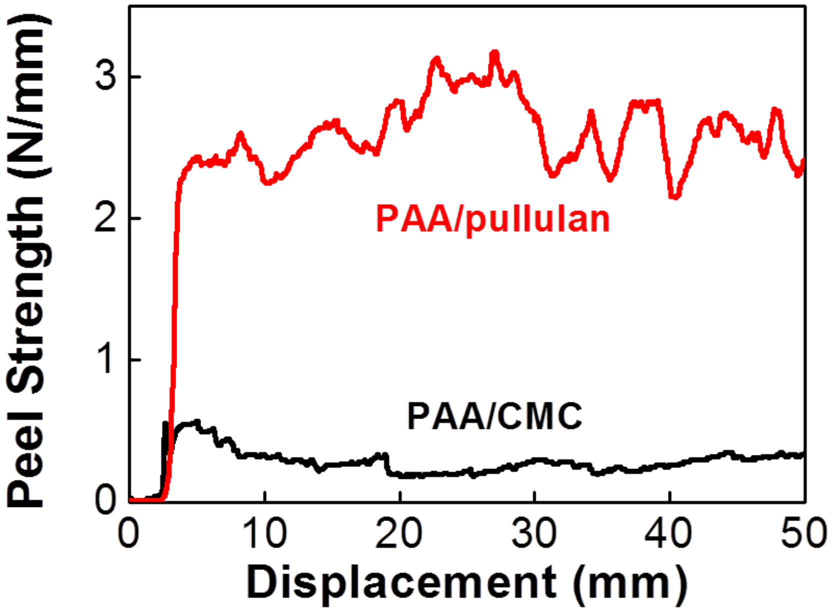

Figure S13 | Adhesion properties of composite electrode films on current collectors. 58 um-thick composite electrodes films on copper current collectors were used for the180 degree adhesive peel strength tests. The composite films based on PAA/pullulan were peeled off from copper current collector by the force which is 7 times larger than that of PAA/CMC-based composite films.

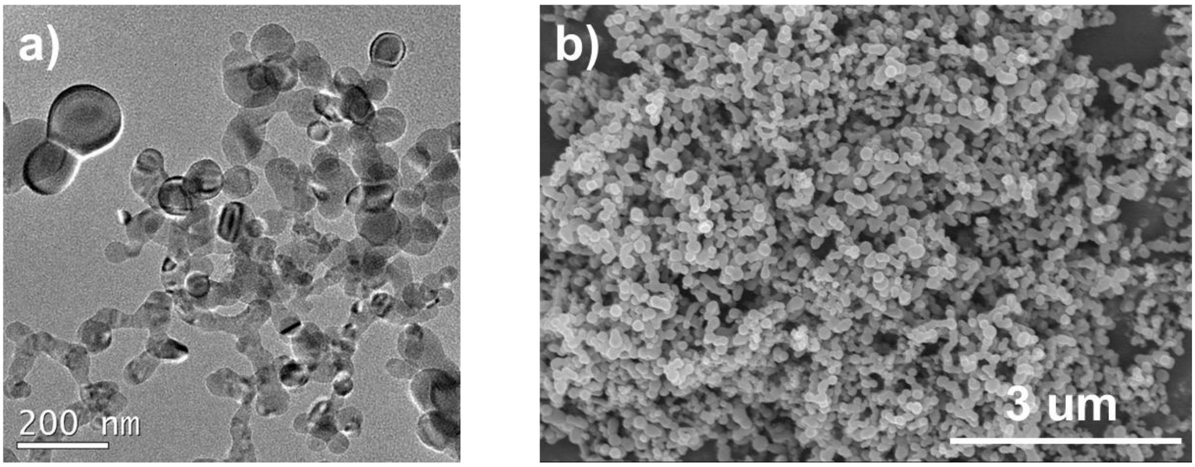

Figure S14 | Morphology of silicon nanoparticle (npSi). a) Transmission electron microscope image. **b**) Scanning electron microscope image.
